# Predicting Cognitive State from Eye Movements

**DOI:** 10.1371/journal.pone.0064937

**Published:** 2013-05-29

**Authors:** John M. Henderson, Svetlana V. Shinkareva, Jing Wang, Steven G. Luke, Jenn Olejarczyk

**Affiliations:** Institute for Mind and Brain and Department of Psychology, University of South Carolina, Columbia, South Carolina, United States of America; University of Leicester, United Kingdom

## Abstract

In human vision, acuity and color sensitivity are greatest at the center of fixation and fall off rapidly as visual eccentricity increases. Humans exploit the high resolution of central vision by actively moving their eyes three to four times each second. Here we demonstrate that it is possible to classify the task that a person is engaged in from their eye movements using multivariate pattern classification. The results have important theoretical implications for computational and neural models of eye movement control. They also have important practical implications for using passively recorded eye movements to infer the cognitive state of a viewer, information that can be used as input for intelligent human-computer interfaces and related applications.

## Introduction

The visual world contains a vast amount of information, but human perception and cognition are capacity-limited: Only a fraction of available information can be processed at any given moment. Efficient perception and cognition therefore requires properly selecting the most relevant information given the current needs of the viewer. The primary way in which this selection takes place is via eye movements. High quality visual information is acquired only from a limited spatial region surrounding the point of fixation. Visual quality falls off rapidly and continuously from the point of fixation into a low-resolution visual surround. We move our eyes about three to four times each second on average via rapid eye movements (*saccades*) to reorient the eyes within the scene. Visual information is only acquired during periods of relative gaze stability (*fixations*) due to saccadic suppression [1]–[3]. Close or direct fixation of a visual object is therefore typically necessary to perceive visual details, to unambiguously determine identity and meaning, and to encode that item into memory. What we see, remember, and understand about a scene is tightly tied to where we look.

Previous research has demonstrated that aggregate eye-movement measures (e.g., average fixation duration and average saccade amplitude) are influenced by viewing task. For example, average fixation duration and saccade amplitude differ in reading compared to scene viewing [4]–[6]. Similarly, average fixation duration and saccade amplitude differ depending on whether a person is searching a scene for a particular item or trying to commit characteristics of that same scene to memory [5], [7]–[10]. Importantly, although mean values of eye movement measures can change as a function of task, the distributions of these values are highly overlapping across tasks, so randomly sampling from these distributions alone cannot distinguish the task that a person is engaged in.

In the present study, we applied multivariate pattern classification to determine whether eye movements code sufficient information about viewing task that the task can be reliably inferred from eye movements. We used multivariate pattern classification to learn and then predict the task that viewers were engaged in based on their eye movements. Multivariate pattern classification methods, referred to as multivariate pattern analyses (MVPA) in the neuroimaging literature, have been used successfully in cognitive neuroscience to infer the content of representations encoded in patterns of cortical activity from functional neuroimaging data [11]–[13]. It has also successfully been applied to eye movement data to classify the viewer and the visual stimulus [14]. We applied multivariate pattern classification here using the same logic to investigate whether the eye-movement record contains sufficient information to permit inferences about the task that a person is engaged in, and by extension, to their underlying cognitive state.

**Figure 1:**
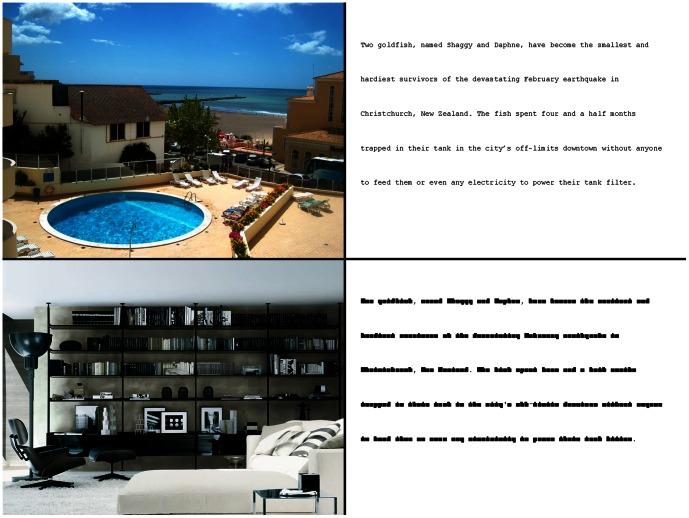
Example stimuli for the A) scene memorization task, B) reading task, C) scene search task and D) pseudo-reading task.

We recorded participants' eye movements while they performed four tasks: scene search, scene memorization, reading, and pseudo-reading (Figure 1). In scene search, participants looked for targets embedded in photographs of real-world scenes. In scene memorization, participants attempted to commit scenes to memory. In reading, participants read paragraphs describing recent news events. In pseudo-reading, participants scanned pseudo-text comprised of geometric shapes instead of letters. We then tested the hypothesis that eye movements are diagnostic of task by training a linear classifier with eye movements recorded from participants engaged in each of the four tasks. This classifier was tested using data not used during training. The tests involved trials within the same session as well as trials from a second session two days later. The goal was to determine if the task that participants were engaged in could be accurately classified from their eye movements.

From a theoretical standpoint, successful classification provides evidence that eye movements are systematically influenced by the viewer's cognitive state, consistent with models emphasizing cognitive control of active vision [15]–[16]. From a practical standpoint, successful classification opens the door to using eye movements as a passive input source for human-computer interfaces and other applications that react to the user's cognitive state.

## Methods

### Participants

Twelve members of the University of South Carolina community participated in the experiment. The experiment was approved by the University of South Carolina Institutional Review Board and all participants gave written informed consent. All participants were native English speakers with 20/20 vision, either corrected or uncorrected, and were naïve with respect to the hypotheses under investigation.

### Apparatus

Eye movements were recorded via an SR Research Eyelink 1000 eye tracker (spatial resolution 0.01°) sampling at 1000 Hz. Participants sat 90 cm away from a 20in monitor, so that scenes and texts subtended approximately 20°×15° of visual angle. Head movements were minimized with chin/head rests. Although viewing was binocular, eye movements were recorded from the right eye. The experiment was controlled with SR Research Experiment Builder software.

### Materials

Stimuli consisted of 196 scene photographs and 140 texts. The texts were taken from online news reports and were 40–60 words long. Scene content varied, and included pictures of both indoor and outdoor environments. Sixty-four scenes contained a 9 pt Arial letter as a search target.

### Procedure

Participants completed two experimental sessions over three days with one intervening day. In both sessions, participants completed the same four tasks. The first task was scene memorization, in which participants viewed scenes (40 per session) in preparation for a later memory test. In the second task, participants read 35 texts per session. In the third task participants searched for small embedded target letters (“L” or “T”) in 48 visual scenes per session. Participants were asked to press a button while fixating on the target when they found it. Trials timed out after 12 seconds. One third of the scenes did not contain a target. The fourth task was pseudo-reading. In this task, participants scanned 35 text-like stimuli per session in which all letters were replaced by block shapes. The pseudo-text was presented in paragraph-like units, and participants were asked to move their eyes through these displays as though they were reading [17]–[19].

All participants completed the tasks in the same order for each session. No participant saw the same stimulus twice. The procedure for both sessions was the same, except that at the end of the first session participants completed a memory test in which they were presented 20 previously viewed scenes and 20 new scenes.

Each trial in each task involved the following sequence. The trial began with a fixation marker at screen center. Once a stable fixation was detected, the stimulus was presented. The participant always viewed the stimulus for 12 seconds before it was removed from the screen, except in search trials when participants could end the trial earlier by pressing the response button.

### Pattern Classification

We used multivariate pattern classification [20]–[21] to investigate whether the task a participant was performing could be identified based on eye movement measures. We addressed this question from two perspectives: (1) whether the task associated with a trial could be identified using training from other trials within the same experimental session (within-session classification), and (2) whether the task performed in one session could be identified based on training from a session conducted on a different day (cross-session classification).

Before classification, fixations longer than 1500 ms were removed from the data, as were fixations and saccades immediately preceding or following blinks. Search trials during which participants located the target were also removed. For each participant, Naïve Bayes classifiers were trained on eight eye movement features capturing eye movement patterns for each trial. As a widely applied classifier, the Naïve Bayes classifier is conceptually simple, less likely to generate over-fitting compared to non-linear classifiers, and effective in various situations even when data do not meet its assumptions [20]–[21]. The features were the mean and standard deviation of fixation duration, the mean and standard deviation of saccade amplitude, the number of fixations per trial, and the three parameters μ, σ, and τ quantifying the shape of the fixation duration distribution with an ex-Gaussian distribution [22]–[24]. The ex-Gaussian distribution is known to change for different eye-movement tasks [25]. The ex-Gaussian distribution was fit to the data from each participant in each trial using QMPE software [26].

The classification was performed in a leave-one-out cross-validation approach to ensure unbiased evaluation of classification performance: in a cross-validation fold, the classifier was trained on all but one trial of data and applied to the left-out test trial to identify its class membership until each trial was classified. Accuracy was the proportion of correctly classified trials. For cross-session classification for each participant, classifiers were trained on data from Day 1 and tested on data from Day 3, and vice versa. Accuracy was the proportion of correctly classified trials. Classification performance was evaluated by comparison to an empirically generated null distribution formed by 2000 classification accuracies from non-informative permutations of labels in the training set [27]. Classification accuracies for each classification model were evaluated at the .05 significance level based on the corresponding null distribution. For within-session classification for each participant, the task associated with a given trial was identified using data from the same session, either Day 1 or Day 3.

## Results

### Within-session classification

**Figure 2:**
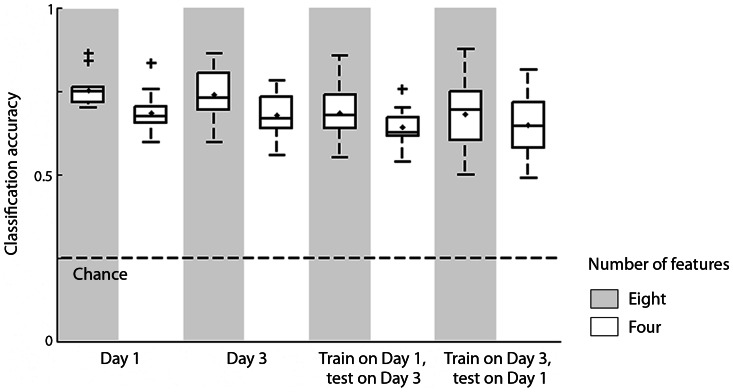
Classification accuracies for identifying one of the four tasks based on eye-movements are shown for different classification models. Accuracies for the 12 participants for each type of classification are summarized in a boxplot. On each box, the central mark is the median, the edges of the box are the 25th and 75th percentiles, the whiskers extend to the most extreme values not considered outliers, and values beyond the 1.5 interquartile ranges are marked with pluses. The mean classification accuracies across the 12 participants are shown as dots.

Classifiers were trained for each participant to identify whether the participant was reading, pseudo-reading, searching scenes, or memorizing scenes. As shown in Figure 2, classification accuracies were reliably above chance for all 12 participants on both Day 1 (M = 0.753, SD = 0.050) and Day 3 (M = 0.739, SD = 0.077). Moreover, classification based only on four features (means and standard deviations of fixation duration and saccade amplitude) also resulted in accuracies significantly above chance (Day 1: M = 0.683, SD = 0.064; Day 3: M = 0.678, SD = 0.064). These results showed that the viewing tasks are highly distinguishable based on eye-movement features in a four-way classification.

### Cross-session classification

The robust successful within-session classifications strongly suggest that task can be reliably identified from eye movements. We also investigated whether task can be identified in a temporally separate session. Classification accuracies (Figure 2) showed that tasks could be identified reliably above chance using eight features for all participants, both when the classifier was trained on Day 1 and tested on Day 3 (M = 0.685, SD = 0.079), and vice versa (M = 0.681, SD = 0.106). Classification accuracies were also reliably above chance with only four features (Day 1 to Day 3: M = 0.640, SD = 0.055; Day 3 to Day 1: M = 0.649, SD = 0.098).

### Classification of scene tasks

**Figure 3:**
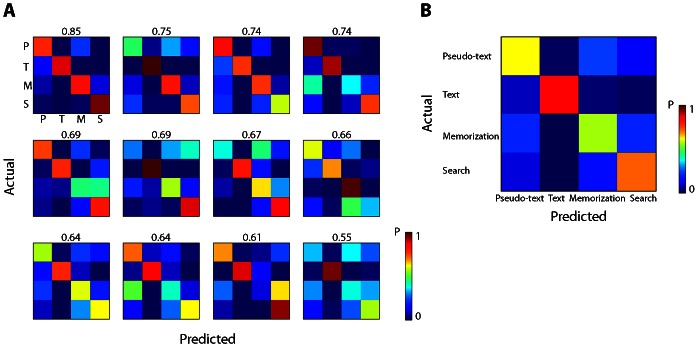
Confusion matrices. Panel A: Confusion matrices for identifying one of the four tasks from one day to another for each participant, ordered by classification accuracies (shown above each matrix). The value of each element denotes the proportion of trials identified as the corresponding label to the total number of trials in the actual category. For example, the first row in a confusion matrix indicates the proportions of all the pseudo-text reading trials that were classified as pseudo-text reading, text-reading, scene memorization, and scene search. A perfect classification results in a confusion matrix with 1 s on the diagonal and 0 s on off-diagonal elements. Panel B: Averaged confusion matrix across the participants.

**Figure 4:**
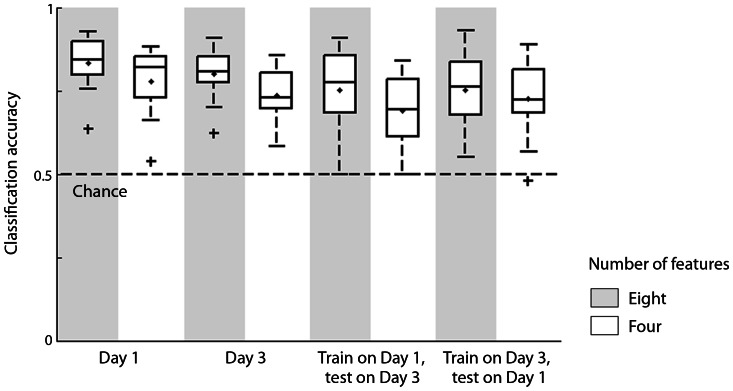
Classification accuracies for identifying two scene-related tasks based on eye movement patterns are shown for different classification models. Accuracies of 12 participants for each type of classification are summarized in a boxplot as in Figure 2.

Examination of confusion matrices (see Figure 3 for an example) revealed that text reading was more accurately identified compared to other tasks for all participants, whereas for some participants memorization and scene search were more similar. We therefore conducted additional classifications on just the two tasks that involved the scene stimuli: search and memorization. This is a more stringent test of task classification from eye movements because the stimuli were identical. Figure 4 shows the overall accuracy in all classifications. In the within-session classification, accuracies were reliably above chance for all of the participants for both Day 1 (M = 0.833, SD = 0.083) and Day 3 (M = 0.800, SD = 0.081). Using only four features also resulted in accuracies significantly above chance (Day 1: M = 0.778, SD = 0.103; Day 3: M = 0.736, SD = 0.083). In the between-session classifications, accuracies were significantly above chance for eight participants when classifiers were trained on Day 1 and tested on Day 3 (M = 0.752, SD = 0.125), and for eight participants when classifiers were trained on Day 3 and tested on Day 1 (M = 0.752, SD = 0.120). Classification accuracies using only four features were significantly above chance for seven participants when trained on Day 1 and tested on Day 3 (M = 0.690, SD = 0.116), and for seven participants when trained on Day 3 and tested on Day 1 (M = 0.724, SD = 0.116).

## Discussion

Participants' eye movements were recorded while they performed four tasks: reading, pseudo-reading, scene search, and scene memorization. A classifier was trained and tested on eye-movement measures to determine whether the tasks that viewers were engaged in could be inferred from their eye-movement behavior. The results demonstrated robust classification accuracy both within a session and across sessions with an intervening day between them. A classifier trained on a set of data and tested on another set of data in the same session reached classification accuracy over 80% compared to a 25% chance baseline. Furthermore, a classifier trained on data collected one day and tested on data collected two days earlier or later produced classification accuracy of about 68% compared to the 25% chance baseline.

These results strongly suggest that eye movements are systematically influenced by the task that a person is engaged in, and by extension, by their cognitive state. This result has implications for understanding eye-movement control from both computational and neural perspectives. In both reading and scene perception, an important theoretical debate focuses on whether eye movements primarily reflect stimulus- and oculomotor-based factors or cognitive factors (in scene perception [16], [28]–[29]; in reading [15], [30]–[31]). The current demonstration that eye movements encode sufficient information for successful classification of viewing task provides new evidence that cognitive processes directly influence eye movements, consistent with theories that adopt a role for top-down control of eye movements in complex tasks (e.g. [15]–[16], [30]–[31]).

It is important to note that the successful classification reported here was not simply based on the general class of the stimulus or on specific stimulus items. First, although classification of reading and picture viewing might in part be based on the general classes of stimuli (text or photographs), successful classification of memorization versus search of photographs could not be due to differences in stimuli. Furthermore, in the case of the cross-session classifications, different stimulus items were used in each of the two sessions (i.e., different texts, pseudo-texts, and photographs), so cross-session classification demonstrated generalization beyond specific stimulus items.

From a practical standpoint, our results suggest that eye movements can be used as a passive, non-invasive source of input to infer a person's cognitive state. The present results operationalize cognitive state in terms of viewing task (e.g., is the user searching for something, reading, memorizing, skimming?), but inferences about other states (e.g., confusion, inattention) might also be possible. In terms of application, given the ubiquity of computers and smart-phones with a user-facing camera, it may be possible to monitor eye movements during use of these devices, determine a viewer's task or current state, and alter the display in a context-sensitive manner based on that state. Similarly, during driving or piloting, a smart car or aircraft might change the information presented on a heads-up display or other output device depending on classification of the operator's state. Similar examples would apply for any condition in which eyetracking is possible. An important advantage of using eye movements rather than button presses or other active input is that they are a natural part of visual and cognitive interaction with the environment, so users do not need to be trained in their use or make any unnatural type of response. These examples illustrate how systems might use eyetracking as input to smart interfaces designed to react to the user's needs.

Note that in the present study we did not include information about where the viewers were looking, but instead only included basic information about general features of their eye movements. This is an important point because in dynamic visual environments where the visual input is constantly changing in uncontrollable ways (e.g., for a car driver, pilot, or otherwise mobile viewer) it is extremely difficult to map eye position onto ever-changing visual objects. However, eye movement features like those used here do not require information about location. It is highly likely that in cases where eye position can be mapped to objects so that the mapping can be included in task classification, classification would be even better.

A potential extension of this work would be to classify a user's cognitive state more generally beyond viewing task. For example, as smart glasses and other integrated eyepieces are developed (e.g., Google Glass), the door opens for recording eye movements continuously and classifying a wide variety of general cognitive states like confusion, concentration, fatigue, alertness, arousal, deception, and so on. Systems could then respond appropriately based on this classification. Again, a benefit of this approach is that eye movements are a natural component of mental and visual activity and therefore do not require an overt response on the part of the user. Eyetracking analysis and classification of cognitive state might take place locally (e.g., in the case of a car, in an on-board computer) or might take place distally through cloud computing by offloading classification to remote servers on a network.

The present study likely underestimates how well eye movements can be used to infer a person's cognitive state. We did not attempt to identify an optimal classifier and it may be that classification could be improved beyond the relatively high levels observed here by using a different classification algorithm. Also, we did not attempt to optimize the set of eye-movement features used in the analyses. Instead, a few common and easily generated eye-movement features were used. Other eye movement features might be more discriminative and provide higher classification accuracy. The present results suggest that investigating alternative classifiers and eye-movement features would be worthwhile. In future work it will also be interesting to determine more generally which other aspects of a participant's cognitive state can be inferred from his or her eye-movement behavior.
